# Substance use disorders among young adults in North-Western Nigeria: descriptive survey of patterns of use

**DOI:** 10.1192/bji.2025.10092

**Published:** 2026-01-28

**Authors:** Charles Marke, Oluwole Jegede, Hesham Mukhtar, Abigail Ojo, Yusuf Boman, Bashir Yakasai, Charles Dike

**Affiliations:** 1 Lecturer, Department of Psychiatry, College of Medicine, Kaduna State University, Kaduna, Nigeria; 2 Assistant Professor, Department of Psychiatry, Yale University School of Medicine, New Haven, Connecticut, USA; 3 Postdoctoral Associate, Department of Psychiatry, Yale University School of Medicine, New Haven, Connecticut, USA; 4 Department of Psychiatry, Yale University School of Medicine, New Haven, Connecticut, USA; 5 Lecturer, Department of Psychiatry, College of Medicine, Kaduna State University, Kaduna, Nigeria; 6 Professor, Department of Psychiatry, College of Medicine, Kaduna State University, Kaduna, Nigeria; 7 Professor, Department of Psychiatry, Yale University School of Medicine, New Haven, Connecticut, USA

**Keywords:** Addictions, substance use disorder, Nigeria, West Africa, epidemiology

## Abstract

**Background:**

Substance use disorder (SUD) is a rapidly growing public health challenge in developing countries across socioeconomic divides. In sub-Saharan Africa, the situation of SUD is particularly concerning and largely unexplored, with projections indicating a worsening trend.

**Aims:**

This study seeks to fill the gap by generating insights into the multifaceted nature of alcohol and drug use disorders among a young adult population in Nigeria.

**Method:**

This is a cross-sectional survey of 192 current students at a university of a metropolitan city in North-Western Nigeria, using the NIDA-Modified ASSIST version 2.0, adapted from the Alcohol, Smoking and Substance Involvement Screening Test.

**Results:**

About half of the participants (49.7%) were heavy drinkers, 36.5% and 56.8% reported past year tobacco smoking and use of prescription drugs for non-medical reasons, but only 7.4% had used illegal drugs daily in the past year. Cannabis and sedatives were the most used substances in the lifetime (56.2% and 47.9%, respectively) and past 3 months (52.4% and 51.1%, respectively). Men had greater odds of substance use in their lifetime (odds ratio 4.167, 95% CI 1.61–10.77; d.f. = 1, *P* = 0.003) and past three months (odds ratio 6.059, 95% CI 2.20–16.69; d.f. = 1, *P* ≤ 0.001), compared with women.

**Conclusions:**

The burden of SUD remains a major public health concern in Nigeria despite existing legislation, regulations and policies in the country. There is an urgent need improve diagnostic, treatment and preventative resources by engaging a massive public health campaign to alert the public of the dangers of SUD.

Substance use disorder (SUD) is a rapidly growing global public health challenge, including low- and middle-income countries, across races and ethnic divides.^1^ The United Nations Office on Drugs and Crime (UNODC) underscores the widespread nature of SUD, reporting that one in every 17 individuals aged 15–64 years worldwide engaged in drug use within the past 12 months.^2^ Although the nature of specific substances used shows some variability across the world, the economic burden and human toll has been consistent, impacting the cost of healthcare, social services, criminal justice resources, productivity, morbidity and mortality.^3^ The annual medical cost associated with SUD in US emergency departments and in-patient settings exceeded $13 billion in 2017,^4^ likely a far cry from the current economic reality, as the country struggles with the fourth wave of the opioid epidemic resulting in over 100 000 deaths per year since the COVID-19 pandemic.^5^

In sub-Saharan Africa, the situation of SUD is particularly concerning and largely unexplored, with projections indicating a worsening trend^6,7^ resulting from the unique demographic characteristics of this region, sociocultural factors, limited resources, poor data availability and limited access to evidence-based treatments. Data on SUD in sub-Saharan is limited; studies are small scale and cross-sectional, mostly addressing substance use rather than SUD among participants; and few studies employ validated diagnostic tools.^7^

However, available studies show a high prevalence of substance use in Nigeria; for example, a study of university students showed a drug use prevalence of 45.7% despite a risk awareness level of 94.6%, with alcohol use being the most at 61.5%.^8^ Two studies among secondary school children reported a wide range of prevalence of substance use of 56% in Northern Nigeria^9^ and 26.3% in Southern Nigeria.^10^ A systematic review of 16 articles in eight sub-Saharan African countries showed a wide range of alcohol use disorder (AUD) prevalence in Nigeria, ranging from 0.1 to 33.2%. Factors associated with AUD included male gender, low income, Catholic religion and the presence of a psychiatric comorbidity.^7^ A large population survey by the UNODC, reported that about 14.4% of the Nigerian population aged between 15 and 64 years old (14.3 million people) had current and continuing substance use, and estimated up to 3 million having at least a form of drug use disorder.^11^

The paucity of national data and lack of systematic reporting of SUD in Nigeria has limited the understanding of the scope of the problem regarding the prevalence of drug use and drug use disorder, which, in turn, has hindered comprehensive clinical, policy and public health solutions to this worsening problem.^10^ Enactment of heavily punitive laws and policies to attempt to combat the rising substance use – including the National Drug Law Enforcement Agency Act, Dangerous Drugs Act and Indian Hemp Act, which criminalise cultivation, possession and trafficking, with sanctions ranging from long prison terms to life imprisonment, asset forfeiture and even the death penalty for trafficking^8,12^ – have been to no avail, as the burden of drug use remains high.

This study seeks to fill a critical knowledge gap by generating comprehensive insights into the multifaceted nature of AUD and SUD among a young adult population in Nigeria. Understanding the pattern of drug use by this population may ultimately serve to build a treatment system that is responsive to the needs of the community. The present study uses a validated instrument to explore patterns of use across a variety of substances, among young adults in a major, diverse metropolitan city in North-West Nigeria. The North-West geopolitical zone of Nigeria is the heartland of Hausa-Fulani culture, with the Hausa as the dominant language and Islam shaping most aspects of social and political life. The region is composed of seven states: Jigawa, Kaduna, Kano, Katsina, Kebbi, Sokoto and Zamfara. The traditional Emirate systems, and Islamic scholarship, are also central to this region, with smaller Christian religious groups (especially in southern Kaduna) adding cultural diversity to the region. There are several (over 30) federal, state and privately owned universities and other higher institutions of learning in this region, like other geopolitical zones in the country.

## Method

This is a cross-sectional study conducted at a major university of a metropolitan city in North-West Nigeria. Participants were adult students enrolled in the 2023–2024 academic year.

This study aimed to recruit a sample of convenience of 200 students in the institution. Based on the research team’s previous experience recruiting participants for research at this institution, we identified sites where students typically gather on the school campus, including lecture halls, cafeterias, relaxation/social centres and hostel lobbies. These sites were targeted to approach potential participants and administer questionnaires. To maximise participation, research assistants who were also students at the institution were trained to administer questionnaires.

### Instruments

Sociodemographic characteristics were obtained for all participants and the NIDA-Modified ASSIST version 2.0, adapted from the World Health Organization (WHO) Alcohol, Smoking and Substance Involvement Screening Test (ASSIST) version 3.0, was used as the main survey instrument.^13^ Participants were initially screened with the NIDA Quick Screen version 1.0, those who marked ‘yes’ to illegal/prescription drugs for non-medical purposes proceeded to the NIDA-modified ASSIST version 2.0.

### Data collection procedure and ethical declaration

The study was conducted between April and June 2024. Although the questionnaires were self-administered, research assistants were available in real time to explain the various aspects of the study, including to obtain signed informed consent, assure participants of data privacy and respond to possible concerns about any items in the questionnaire. Signed consent was obtained, witnessed and recorded by the research assistant as required by the institutional review board. The questionnaires were presented to participants in English. Participants were not compensated for their participation in the study. Participants were included only if they met inclusion criteria: adult students, aged at least 18 years and enrolled in the current academic year at the university.

The authors assert that all procedures contributing to this work comply with the ethical standards of the relevant national and institutional committees on human experimentation and with the Helsinki Declaration of 1975, as revised in 2013. All procedures involving human patients were approved by the Ethical Committee of the Kaduna State University, Kaduna, Nigeria (approval number KASU/HEC/2024/0001).

### Statistical analysis

All analysis was done with the Statistical Package for the Social Sciences for Windows (IBM SPSS Statistics, Version 31 (IBM Corporation, Armonk, New York, USA; https://www.ibm.com/products/spss-statistics)). Microsoft Excel (Microsoft Excel for Windows (Microsoft Corporation, Redmond, Washington, USA; https://www.microsoft.com/microsoft-365/excel)) was initially used for data entry, coding and editing. Sociodemographic information was described with descriptive statistical methods, including means, percentages and standard deviation.

Logistic regression was used to determine the association between gender and four outcome measures: (a) lifetime substance use; (b) substance use in the past 3 months; (c) task failure in the past 3 months and (d) health, social, legal or financial problems in the past 3 months. A separate logistic regression model was conducted for each of the outcome variable, with gender (male versus female) as the independent variable. In all models, female gender was used as the reference category. Logistic regression was applied to find out the odds ratio for each of the dependent measure between the two genders. Results were reported as odds ratios with corresponding *P*-values and 95% confidence intervals for each outcome.

## Results

The study recruited 200 participants, but only 192 participants completed the final surveys and were enrolled in the final study sample. Most of the participants were aged between 18 and 30 (70.8%) years, and were female (54.7%).

### Initial screen

The initial survey was conducted with the NIDA Quick Screen for alcohol, tobacco, prescription and illegal drugs use in the past year (Table 1). Alcohol screening was based on drinking five or more drinks a day (for men) and four or more drinks a day (for women), and showed that 49.7% of the participants (both men and women) had a pattern of heavy drinking in the past year, 8.4% had a pattern of daily heavy drinking and 12% had a pattern of weekly heavy drinking. For tobacco screening, of 122 respondents, 36.5% were smokers in the past year, only 6.8% smoking daily and 2.6% were smoking tobacco and tobacco products weekly. With regards to the use of prescription drugs for non-medical reasons, 108 participants (56.8%) responded that they had never used any prescription drugs for non-medical reasons, whereas only 7.4% reported daily use of any prescription drugs for non-medical reasons in the past year. Finally, 53.4% had never used illegal drugs, whereas 7.4% used illegal drugs daily in the past year. Participants who answered yes to using prescription drugs for non-medical reasons or illegal drug use proceeded to complete the NIDA-Modified ASSIST version 2.0.

**Table 1:** Past year alcohol, tobacco and illegal drug use (NIDA Quick Screen) (*n* = 192)

	*n* (%)
Alcohol	96 (50.0)
Tobacco	70 (36.5)
Prescription drugs	84 (43.8)
Illegal drugs	91 (47.4)

### NIDA-modified ASSIST version 2.0

#### Lifetime and 3-month substance use

Lifetime and 3-month substance use (including once monthly, weekly or daily use over the 3-month period) are as reported in Tables 2 and 3. The most reported lifetime substance used was sedatives at 56.2%, followed by cannabis at 47.9%. The same pattern was observed for 3-month substance use for sedatives and cannabis (52.4 and 51.1%, respectively). Hallucinogens were the least common over the lifetime and 3-month substance use among the participants, at 13% and 16.9%, respectively. Of the entire population, 75.6 and 83.1% had never used cocaine and hallucinogens in the past 3 months. Further, 20% of the population smoked cannabis daily (or almost daily), compared with 47.9% who reported a lifetime use.

**Table 2:** Lifetime history of substance use

Substance	Yes		No		*N*
	*n*	%	*n*	%	
Cannabis	79	47.9	86	52.1	165
Cocaine	30	18.8	130	81.3	160
Prescribed amphetamines	31	19.5	128	80.5	159
Methamphetamine	43	27.0	116	73.0	159
Inhalants	36	22.8	122	77.2	158
Sedatives	95	56.2	74	43.8	169
Hallucinogens	21	13.0	140	87.0	161
Street opioids	40	25.2	119	74.8	159
Prescribed opioids	29	18.0	132	82.0	161
Other	68	50.0	68	50.0	136

**Table 3:** Substance use in the past 3 months

Substances	Never		Once/twice		Monthly		Weekly		Daily or almost daily		*N*
	*n*	%	*n*	%	*n*	%	*n*	%	*n*	%	
Cannabis	66	48.9	25	18.5	8	5.9	9	6.7	27	20.0	135
Cocaine	90	75.6	9	7.6	10	8.4	4	3.4	6	5.0	119
Prescribed amphetamine	92	75.4	10	8.3	10	8.2	8	6.6	2	1.6	122
Methamphetamine	77	64.7	17	14.3	11	9.2	7	5.9	7	5.9	119
Inhalants	90	75.6	10	8.4	6	5.0	8	6.7	5	4.2	119
Sedatives	68	47.6	29	20.3	18	12.6	21	14.7	7	4.9	143
Hallucinogens	98	83.1	7	5.9	9	7.6	0	0.0	4	3.4	118
Street opioids	79	67.5	12	10.3	10	8.5	8	6.8	8	6.8	117
Prescribed opioids	91	77.1	8	6.8	7	5.9	7	5.9	5	4.2	118
Others	62	53.4	15	12.9	16	13.8	15	12.9	8	6.9	116

#### Lifetime substance use by gender

In our sample, 71 (81.6%) out of 87 men and 71 (67.6%) out of 105 women reported substance use in their lifetime (Table 4). The most used substance in men was cannabis (*n* = 49/87, 56.3%), compared with women, who most commonly used sedatives (*n* = 49/105, 46.7%) (Table 4). The least commonly used substances in males were hallucinogens (*n* = 11, 12.6%), but for women, hallucinogens (*n* = 10, 9.5%) and street opioids (*n* = 10, 9.5%) were the least commonly used substances in their lifetime. Logistic regression was performed on male and female substance use, with lifetime substance use as the dependent variable and gender as the independent variable (women were used as the reference category). The model was highly significant (*χ* ^2^ = 10.42, d.f. = 1, *P* = 0.001), revealing that men had greater odds of substance use in their lifetime compared with females (odds ratio 4.167, 95% CI 1.61–10.77; d.f. = 1, *P* = 0.003).

**Table 4:** Lifetime (mean = 87, F = 105) and past 3-month substance use by gender

	Lifetime		3-months	
	Male	Female	Male	Female
Cannabis	49	30	45	24
Cocaine	13	17	14	15
Prescription stimulant	14	17	16	14
Methamphetamine	29	14	28	14
Inhalant	20	16	17	12
Sedative	46	49	39	36
Hallucinogen	11	10	11	9
Street opioid	30	10	29	9
Prescription opioid	14	15	13	14
Other	36	32	28	26

#### Past 3-month substance use by gender

Of 87 men, 66 (75.9%) reported substance use in the past 3 months, and of 105 females, 61 (58.1%) reported substance use in the past 3 months. Like the lifetime pattern, for men, the most used substance in the past 3 months was cannabis (*n* = 45/87, 51.7%), but for women, sedatives were the most used substance (*n* = 36/105, 34.3%) (Table 4). For men, the least used substances were hallucinogens (*n* = 11, 12.6%), but for women, it was hallucinogens (*n* = 9, 8.6%) and street opioids (*n* = 9, 8.6%) (Table 4). Logistic regression was applied with past 3-month substance use as the dependent variable and gender as the independent variable (women used as the reference category). The resulting model was highly significant (*χ* ^2^ = 15.844, d.f. = 1, *P* ≤ 0.001), revealing that men had higher odds of past 3-month substance use compared with women (odds ratio 6.059, 95% CI 2.20–16.69; d.f. = 1, *P* ≤ 0.001).

#### Past 3 months task failure in men and women

Table 5 describes the failure to perform daily tasks (normally expected of a person) in the past 3 months due to substance use in men and women. Fifty-six men (64.4%) failed to perform daily tasks in the past 3 months, compared with 48 (45.7%) women. Cannabis led to the highest frequency of task failure in men (*n* = 42/87, 48.35%) and sedatives led to the highest frequency of task failure in women (*n* = 27/105, 25.7%). The substances that led to the least task failure were hallucinogens (*n* = 10/87, 11.5%) in men and street opioids (*n* = 8/105, 7.6%) in women. Logistic regression was performed with past 3-month task failure as the dependent variable and gender as the independent variable (women were used as the reference category). The resulting model was highly significant (*χ* ^2^ = 10.677, d.f. = 1, *P* = 0.001), revealing that men had greater odds of task failure than women in the past 3 months (odds ratio 3.759, 95% CI 1.62–8.72; d.f. = 1, *P* = 0.002).

**Table 5:** Failure to perform normal daily tasks in the past 3 months (mean = 87, F = 105)

	Male	Female
Cannabis	42	19
Cocaine	12	14
Prescription stimulant	12	10
Methamphetamine	25	11
Inhalant	19	12
Sedative	29	27
Hallucinogen	10	9
Street opioid	26	8
Prescription opioid	13	12
Other	26	22

#### Health, social, legal or financial problems in the past 3 months

The different substances causing health, social, financial or legal problems in men and women are as described in Table 6. Of 87 males, 58 (66.7%) experienced at least one of the mentioned issues in the past 3 months, compared with only 45 (42.9%) out of 105 women. Cannabis (*n* = 43/87, 49.4%) was the leading cause of any legal, health, financial or social problems for men, and sedatives (*n* = 27/105, 25.7%) were the leading cause for women. For men, the least reported substances causing these issues were hallucinogens, cocaine and prescription opioids (*n* = 13/87, 14.9%); for women, the least reported substances were hallucinogens and street opioids (both at *n* = 9/105, 8.6%). Logistic regression was performed with any health, legal, financial or social issue as the dependent measure and gender as the independent measure (women were used as the reference category). The resulting model was highly significant (*χ* ^2^ = 14.654, d.f. = 1, *P* ≤ 0.001), revealing that men had greater odds of getting into any financial, social, legal or health issue compared with women in the past 3 months (odds ratio 4.382, 95% CI 1.96–9.81; d.f. = 1, *P* ≤ 0.001).

**Table 6:** Health, social, legal or financial problems in the past 3 months (mean = 87, F = 105)

	Male	Female
Cannabis	43	19
Cocaine	13	15
Prescription stimulant	15	11
Methamphetamine	26	14
Inhalant	20	14
Sedative	30	27
Hallucinogen	13	9
Street opioid	27	9
Prescription opioid	13	13
Other	23	22

#### Loss of control, functional and social consequences

Based on some of the DSM-5-TR criteria for SUD, participants were asked a series of questions, including the following: In the past 3 months (Table 7), have you had a strong desire/urge to use? How often has your use led to health, social, legal or financial problems? How often have you failed to do what was normally expected of you because of your drug use? Have your friend or relative or anyone else ever expressed concerns about your drug use? Have you tried and failed to control, cut down or stop drug use?

**Table 7:** Criteria for substance use disorder (positive at least once in past 3 months)

	A *n* (%)	B *n* (%)	C *n* (%)	D *n* (%)	E *n* (%)
Cannabis	40 (54.2)	62 (46.6)	61 (47.6)	64 (47.4)	62 (47.3)
Cocaine	29 (25.2)	28 (23.7)	26 (22.8)	30 (25.2)	29 (25.0)
Prescription stimulant	25 (21.6)	26 (22.0)	22 (18.6)	23 (19.2)	24 (20.7)
Methamphetamine	38 (32.8)	40 (33.9)	36 (31.6)	37 (31.9)	35 (31.0)
Inhalant	31 (25.7)	34 (29.1)	31 (27.8)	30 (25.4)	32 (27.1)
Sedative	64 (54.7)	57 (44.2)	56 (41.9)	60 (43.8)	59 (44.7)
Hallucinogen	20 (15.9)	22 (18.6)	19 (15.8)	20 (16.8)	18 (15.7)
Street opioid	37 (31.9)	36 (30.5)	34 (28.1)	33 (27.7)	36 (31.6)
Prescription opioid	26 (22.4)	26 (22.2)	25 (20.2)	26 (21.8)	23 (20.4)
Other	55 (51.4)	45 (42.1)	48 (46.2)	49 (45.0)	43 (40.6)

A, Strong desire/urge to use. B, Use led to health, social, legal or financial problems. C, Failure to do what is expected. D, Friend or family experienced concern about use. E , Tired and failed to control, cut down or stop use.

Participants who used cannabis in the past month were the most to frequently report ‘strong desire/urge to use’, at 54.7%; those who used cannabis were the most likely to report that their use led to health, social, legal or financial problems, at 46.6%, followed by those who reported sedative use, at 44.2%; those who used cannabis were also the most likely to report failure to do what is expected, at 47.6%, followed by ‘other’ substances and sedatives, at 46.2 and 41.9%, respectively. The same pattern was observed for friends or family expressing concern about drug use, with cannabis, other substances and sedatives at the highest frequencies (47.4, 45 and 43.8%, respectively). Finally, the pattern remained the same when we considered failure to control, cut down or cut use: the highest frequencies were for participants who used cannabis, sedative and ‘other’ substances in the past 3 months, at 47.3, 44.7 and 40.6%, respectively (Table 7).

#### Injection drug use

Injection of any drug for non-medical use was reported in Table 8. As shown, 68.1% of respondents reported that they had never used any drug via the parenteral route. Of those who reported a lifetime injection use of any drugs (31.9%), 21% had not used in the past 3 months, whereas 10.9% had used in the past 3 months.

**Table 8:** Injection of any drug for non-medical use

Have you ever used any drug by injection?			Yes				
	Never		Not in past 3 months		In past 3 months		
	*n*	%	*n*	%	*n*	%	*N*
	81	68.1	25	21.0	13	10.9	119

## Discussion

This present study is a cross-sectional survey of the pattern of substance use among university students in a major metropolitan city in Northern Nigeria. The literature of patterns of substance use in Nigeria and in sub-Saharan Africa is limited and sparse. This study extends previous literature in various ways: (a) its use of standardised and validated instrumentation to gather data moves beyond only snapshots of substance use, to allow us to also establish SUD; (b) the population of young adults in North Nigeria is novel, as this population have not been the focus of prior limited research; and (c) the survey expands the list of substances to include those that were not traditionally included in previous literature, including inhalants, as represented in the survey utilised.

The initial screen based on the National Institute of Alcohol Abuse and Alcoholism (NIAAA) daily drinking standards showed that about half (50%) of the entire population met the criteria for heavy alcohol use. This is consistent with other studies from Nigeria that reported high lifetime use of alcohol; for example, Gureje and colleagues reported a lifetime prevalence of 56%.^14^ However, our results are higher than a previous large cross-sectional study of individuals aged 15–64 years across all six geopolitical zones in the country, which found a lifetime prevalence of 39%.^15^ Compared with the studies by Gureje et al^14^ and Adamson and Adebowale,^15^ we found a higher incidence of daily tobacco (and tobacco products) smoking at 6.8% versus 3 and 4.1%, respectively; this is likely because of the geographic specificity (this area has been shown to use tobacco more than other areas of the country), urbanicity and university age of our respondents. Of note, tobacco and tobacco products refer to cigarettes, e-cigarettes, nicotine pouches, waterpipe tobacco, cigars, cigarillos, heated tobacco, roll-your-own tobacco, pipe tobacco, bidis and kreteks, and smokeless tobacco products.^16^

Our study also shows consistency with previous gender-based substance use research, across all metrics measured, as we found that men were significantly more likely to use substances in general, with cannabis as the most used substance.

Our study uncovered a surprising and disturbing use of injectable drugs. We found that about 30% of our population reported a lifetime history of injection drug use (10.9% had used in the past 3 months and 21% had not used in the past 3 months). The significance of this find is robust given the conservativeness of North Nigeria and the health hazards associated with parenteral drug use. For example, this is underscored by a study of the prevalence of infectious diseases among injection drug users in Nigeria: 74% of the population screened reported injecting drugs in the past month, with estimated hepatitis B, hepatitis C, HIV, syphilis, gonorrhoea and chlamydia prevalences of 7.8, 7.7, 0.9, 1.9, 0 and 3.7%, respectively.^17^ Our results are much higher than a previous study of secondary school students that reported intravenous drug use at 0.8%:^18^ the difference is likely because of the age of our participants and that our study included all injection routes (intravenous and intramuscular).

A secondary analysis of data from the Nigerian Epidemiological Network of Drug Use for the years 2017 and 2018 showed that 5.1% of the population were injection drug users, a majority (78.7%) of which were currently injecting at treatment entry. Opioids (tramadol, codeine, morphine) were the most commonly injected drugs.^19^ Of note is the deficient state of SUD education in Nigeria. In a study among prescribers, although 85.7% endorsed the need for medication for opioid use disorder treatment in Nigeria, the majority (65.3% of prescribers) had no previous experience, 74.2% had no training in prescribing and 68.7% had no knowledge of existing guidelines for treatment.^20^

This study is not without its limitations: this is a cross-sectional study of university-aged individuals on campus. Although this is an important demographic reporting highest prevalence^21–23^ of substance use, caution must be exercised not to generalise our results to other populations. Further limiting the generalisability of the results of this study is the non-random sampling technique of convenience (and small) sample size, which is not representative of the population studied. In addition, we have only presented descriptive data and some associations, which preclude causality.

Our results also show an interesting discrepancy among men (Table 4): 3-month prevalence was slightly higher than lifetime prevalence, at 14 versus 13 (cocaine) and 14 versus 16 (prescription stimulant). In interpreting this prevalence estimates, it is important to note that apparent anomalies such as a higher short-term prevalence (e.g. past 3-month or past-year) compared with lifetime prevalence have been observed in national surveys. For example, the 2024 National Survey on Drug Use and Health (NSDUH) Annual National Report describes that field testing of revised prescription drug items in 2012 and 2013 yielded higher past-year misuse rates, but lower lifetime misuse rates relative to the main survey at the time (p. 59). In other words, the wording emphasis on past-year misuse likely resulted in underreporting of lifetime misuse. As in our present study, this pattern likely reflects a measurement artifact rather than a true epidemiologic phenomenon, arising from a combination of recall bias and questionnaire design effects.^24^

Furthermore, because of limited data availability, we were unable to adjust the logistic regression models for potential confounding variables such as age, ethnicity and others. As a result, gender was the only independent variable included in each model. Finally, it is interesting to note that North Nigeria is a conservative society, a fact that may impact the responses provided by participants, theoretically causing underreporting. However, our results show an unexpectedly high prevalence of alcohol and drug use disorders in this cohort. One possible reason for this is a sampling bias, as the cohort consisted largely of university students from various parts of the country, who may have been socialised differently from local northerners.

Future directions of further research including larger and longitudinal studies are required to better evaluate and characterise the epidemiology of SUD in Nigeria and in sub-Saharan Africa.

In conclusion, the burden of SUD remains a major public health concern globally and specifically in Nigeria, despite existing laws, government policies and regulations in the country.^21^ The problem is compounded by the resources needed to engage a massive public health campaign to alert the public of the dangers of SUD. Coupled with the relative lack of technical competence, training and expertise to provide much needed treatment, the situation is dire indeed, and likely to get worse without tight regulation of the influx of illicit substances and focused attention on care at all levels of education and age, and for all genders, in terms of preventative efforts and culturally tailored evidence-based interventions.

## Data Availability

The data for this study is available from the lead author if requested.
